# The prevalence of Lynch syndrome in women with endometrial cancer: a systematic review protocol

**DOI:** 10.1186/s13643-018-0792-8

**Published:** 2018-08-16

**Authors:** Neil A. J. Ryan, Dominic Blake, Marcus Cabrera-Dandy, Mark A. Glaire, D. Gareth Evans, Emma J. Crosbie

**Affiliations:** 1 Division of Cancer Sciences, Faculty of Biology, Medicine and Health, University of Manchester, St Mary’s Hospital, Manchester, UK; 2 Division of Evolution and Genomic Medicine, Faculty of Biology, Medicine and Health, University of Manchester, St Mary’s Hospital, Manchester, UK; 3 The Northern Gynaecological Oncology Centre, The Queen Elizabeth Hospital, Gateshead, UK; 4 Royal Blackburn Hospital, Lancashire Hospitals NHS Trust, Haslingden Road, Blackburn, UK; 5 Tumour Genomics and Immunology Group, The Oxford Centre for Cancer Gene Research, Wellcome Trust Centre for Human Genetics, University of Oxford, Oxford, UK; 6 Manchester Centre for Genomic Medicine, St Mary’s Hospital, Manchester University NHS Foundation Trust, Manchester Academic Health Science Centre, Manchester, UK; 7 Department of Obstetrics and Gynaecology, St Mary’s Hospital, Manchester University NHS Foundation Trust, Manchester Academic Health Science Centre, Manchester, UK; 8 Gynaecological Oncology Research Group, Division of Cancer Sciences, University of Manchester, 5th Floor Research, St Mary’s Hospital, Oxford Road, Manchester, M13 9WL UK

**Keywords:** Lynch syndrome, Endometrial cancer, Systematic review, Prevalence, Immunohistochemistry, Microsatellite instability, Next generation sequencing

## Abstract

**Background:**

Lynch syndrome is the most common inherited cancer syndrome, which predisposes individuals to a number of different cancers, principally colorectal and endometrial cancer. The early diagnosis of Lynch syndrome enables colorectal surveillance, which has been shown to save lives through the detection and removal of premalignant polyps and earlier detection of invasive disease. Endometrial cancer, which is often the sentinel cancer in women, provides an opportunity to diagnose Lynch syndrome and thus enable colorectal surveillance as well as the cascade testing for Lynch syndrome in other family members. These potential benefits have led to a call for the universal screening of women with endometrial cancer for Lynch syndrome, a practice that is now commonplace in colorectal cancer. Healthcare providers and clinicians are however restricted by insufficient knowledge about the prevalence of Lynch syndrome in women with endometrial cancer, with estimates varying as widely as 1–10%. The aim of this study is to perform a systematic review with a meta-analysis of the current literature base in order to estimate the prevalence of Lynch syndrome among women with endometrial cancer to inform this discussion.

**Methods:**

Medline, Embase, Cochrane Central Register of Controlled Trials (CENTRAL), Cochrane Methodology Register, NHS Health and Technology Assessment Database and the Web of Science will be systematically searched for relevant studies via the Ovid platform. Two authors will review the titles and abstracts independently, with discrepancy settled by a third author. Data extraction will be completed to record demographic, pathological and clinical data, as well as the diagnostic methods used for estimating the prevalence of Lynch syndrome in women with endometrial cancer. Bias will be assessed and recorded using the Newcastle-Ottawa Scale and that of the International Cochrane Collaboration. Dependent on the heterogeneity of the data, we aim to produce a cumulative incidence in addition to subgroup analyses as to investigate secondary outcomes.

**Discussion:**

The aim of this systematic review is to provide a robust estimate of the prevalence of Lynch syndrome in women with endometrial cancer. This will enable resource allocation and decision-making regarding the appropriateness of screening all women, or certain women, with endometrial cancer for Lynch syndrome. Such a policy could enable the earlier diagnosis of Lynch syndrome in women and, through the application of colorectal cancer surveillance, improve their survival outcomes.

**Systematic review registration:**

This systematic review has been registered on PROSPERO (ref CRD42017081707).

## Background

Lynch syndrome is the most common inherited cancer syndrome [1]. Those with Lynch syndrome have a compromised mismatch repair system (MMR), which, after secondary gene knockout, leads to a hypermutated phenotype caused by numerous errors in replicated dinucleotide repeats. This phenomenon is referred to as microsatellite instability (MSI) [2]. Mutations in tumour suppressor genes lead to unfaithful genomic replication, which increases the risk of carcinogenesis. Lynch syndrome-associated cancers have defective DNA repair capacity through loss of their MMR proteins [3]. These features allow tumours to be screened and triaged into those that may have arisen as a consequence of Lynch syndrome from others that have not. Women with Lynch syndrome have a cumulative lifetime risk of endometrial cancer of up to 50% [4]. What is less clear, however, is the prevalence of Lynch syndrome in women with endometrial cancer.

A multitude of studies have attempted to define the prevalence of Lynch syndrome in selected and unselected endometrial cancer cohorts. Most have included a small number of participants, but there are a few with cohorts of more than 500 patients [5–10]. The methods used to screen endometrial tumours for Lynch syndrome have varied considerably across different studies and many have failed to complete all tests on all eligible tumours or patients. This has led to wide estimates of the prevalence of Lynch syndrome in women with endometrial cancer of between 1 and 10% [11, 12].

Recently there have been calls for the universal screening of individuals newly diagnosed with colorectal or endometrial cancer for Lynch syndrome [13–16]. Although clinically prudent, this will place extra demands on the limited assets available to global healthcare systems. In order to plan and provide the appropriate levels of expenditure and resources to implement screening for Lynch syndrome, healthcare leaders need an accurate estimate of the true prevalence of Lynch syndrome in women with endometrial cancer. Furthermore, they need to have accurate data on the likely effect of utilising different diagnostic modalities on this estimate. These data are available in the context of colorectal cancer [17]. They remain undefined in endometrial cancer leading to a dichotomy of care whereby those with colorectal cancer are increasingly screened for Lynch syndrome whereas women with endometrial cancer are not [13]. This speaks to a gender equality issue that research needs to address.

The aim of this systematic review will be to provide an overall composite incidence of Lynch syndrome-associated endometrial cancer. This will be explored within the context of the selection criteria, age distribution and diagnostic methods that have been used in different studies. Furthermore, we will aim to provide subgroup analysis depending on the geographical region in which the women were recruited from. The reporting of the protocol is in accordance with the Preference for Systematic Reviews and Meta-analysis Protocols (PRISMA-P) 2015 statement and the International Cochrane Collaborations guidance [18, 19].

## Methods and design

### Objectives

The overall aim of the systematic review will be to determine the prevalence of Lynch syndrome in women with endometrial cancer. This will be achieved by an analysis of proportionality and will look to several disease states as defined below.

### Definitions

For the purpose of this systematic review, Lynch syndrome will be defined as a confirmed germline pathogenic mutation of a MMR gene (*MLH1*, *PMS2*, *MSH2* and *MSH6*) or *EpCAM*, which leads to an epigenetic silencing of *MSH2* [20], and the constitutional methylation of *MLH1* [21]. We will also consider variants of unknown significance described as pathogenic by the study authors as Lynch syndrome.

‘Lynch-like’ will be defined as a tumour with MLH1 or PMS2 protein loss on IHC due to epigenetic silencing of *MLH1* through somatic promoter region methylation. We will further consider tumours with pathogenic mutations in one of the MMR genes at a somatic level as ‘Lynch-like’.

MMR deficient tumours will be defined as tumours in which MLH1, MSH2, MSH6 or PMS2 expression has been lost at protein level, but this has not been defined as either somatic or germline due to the absence of any germline mutational analysis or methylation testing.

MSI will be defined as tumours with molecular evidence of a microsatellite-high (MSI-H) state, which, as per the National Cancer Institute definition, is any cancer where two or more loci (of a five-loci panel) are unstable [22].

This systematic review seeks to define the prevalence of Lynch syndrome in women with endometrial cancer by determining the proportionality. That is, the proportion of endometrial tumours that are associated with Lynch syndrome, defined as the fraction of the total number of endometrial cancers in a given cohort that have Lynch syndrome. This is summarised by the following equation:

$$ \widehat{p}=x/n $$

where ‘*p*’ is the proportion, ‘*x*’ is the number of cases with Lynch syndrome and ‘*n*’ is the total number of endometrial cancers.

In some studies, not all samples that should have been tested for Lynch syndrome underwent appropriate testing. In this scenario, we will devise an estimate of the overall proportion using the standard error of the sample proportion, which is summarised by the following equation:

$$ \sqrt{\frac{\widehat{p}\left(1-\widehat{p}\right)}{n}} $$

### Types of intervention

All current methodologies used in the molecular diagnosis of Lynch syndrome, Lynch-like syndrome and MSI-H will be included as the interventions. These include genomic germline or somatic sequencing for MMR mutations, immunohistochemical analysis of tumour MMR protein expression, polymerase chain reaction (PCR)-based measures of MSI and measures of bisulphate conversion-based quantitative DNA methylation analysis.

### Types of study

All studies that employ the stated interventions with the purpose of defining the prevalence of Lynch syndrome in women with endometrial cancer will be included. We will report the prevalence of MMR deficient, Lynch-like, MSI and Lynch syndrome-associated endometrial tumours. We will include any study that has applied the following methods to endometrial cancers for the purpose of determining the prevalence of Lynch syndrome in women with endometrial cancer:

- Immunohistochemistry to measure tumour MMR protein expression
- Microsatellite instability analysis
- Methylation
- Germline mutation analysis
- Somatic mutation analysis

The use of the B-Raf protein as a proxy for methylation is not substantiated in endometrial cancer and therefore will not be included in this systematic review [23].

**Fig. 1:**
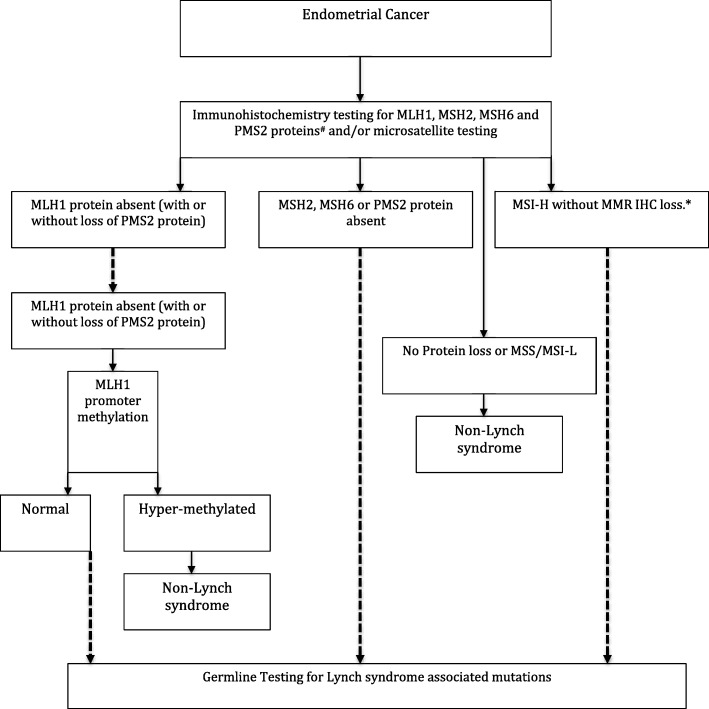
An outline of the potential diagnostic schema used in studies that aim to define the prevalence of Lynch syndrome in endometrial cancer. Dashed lines indicate diagnostic best practice. Abbreviations: MSS, microsatellite stable; MSI-H, microsatellite instability-high; MSI-L, microsatellite instability-low; MMR, mismatch repair; IHC, immunohistochemistry

These tests may have been used in isolation, in combination or in a progressive application. An example of progressive application is given in Fig. 1.

Only studies that include at least 20 endometrial cancers will be included. Studies including fewer than 20 endometrial cancers are unlikely to provide a useful estimate of the true proportion of Lynch syndrome in the endometrial cancer population.

We will include studies of unselected and selected endometrial cancers. Studies that pre-select their endometrial cancer cohort, for example, based on age, clinico-pathological parameters or family history will be included; however, these will be subject to subgroup analysis. In addition, studies that have not tested for all four MMR proteins or the accepted five agreed microsatellite markers will undergo post hoc subgroup analysis to ensure they do not skew the results.

### Types of participant

The participants will be women with endometrial cancer. No restriction will be placed on age, ethnicity or any other demographics. Study setting will be restricted to secondary care.

### Types of outcome

The primary outcome will be the prevalence of Lynch syndrome, Lynch-like, MSI-H or MMR-deficient endometrial cancer as defined by the molecular diagnostic strategy. The methods of molecular testing will be recorded.

In addition, if cases are pre-selected, the conditions for pre-selection will be recorded. Other secondary outcome measures will include the country of origin, exclusion criteria, number of subjects, histopathology data and mutated gene/specific protein lost.

### Literature search

Studies will be identified through an electronic biographical database search, which will include published abstracts as well as complete articles. This search will be devised in liaison with a subject specialist librarian. Trial registers will not be searched as these studies will not have concluded, and therefore, they will not be in a position to define the prevalence of endometrial cancer related to Lynch syndrome. This initial search will be supplemented by primary reference list searches of all the studies selected for full paper review.

### Electronic bibliographical databases

The following electronic bibliographical databases will be searched: Medline, Embase, Cochrane Central Register of Controlled Trials (CENTRAL), Cochrane Methodology Register, NHS Health and Technology Assessment Database and Web of science via the Ovid platform. The grey literature and non-electronic literature will not be included in the preliminary search. The search strategy will aim to include the widest number of studies in the primary review. As such associated Medical Subject Headings (MeSH) will be used. Searches will be limited to the English language, human adults only and female subjects. However, there will be no restriction on the date of publication. The search will be re-run immediately prior to the analysis so that any new studies can be reviewed and, if inclusion criteria are met, included in the study.

A draft search criteria are:

1. Colorectal Neoplasms, Hereditary Nonpolyposis/
2. lynch syndrome.mp.
3. 1 or 2
4. Endometrial Neoplasms/
5. endometrial cancer.mp.
6. 4 or 5
7. 3 and 6
8. limit 7 to (english language and female and humans)
9. from 8 keep 1–2,4

### Study screening and selection

The results from these searches will be combined. The titles and abstracts will be collated in a spreadsheet template downloaded from http://libguides.sph.uth.tmc.edu/systematic-review-guidance. Duplicates will be removed with the use of Endnote X7 (Thompson Reuters, New York, NY, USA). All titles and abstracts will be initially screened independently by two authors. Conflicts will be resolved by a third-party consultation. Where unanimous agreement cannot be reached, a senior author will make the definitive decision. Those identified as meeting the inclusion criteria will undergo full-article review and data extraction. Inclusion criteria are defined above.

### Data extraction

A data collection form will be designed as to ensure the complete capture of all the primary and secondary outcome data points. This will be pre-piloted. Missing data will be requested by email to the identified corresponding author. The data to be extracted is detailed above.

### Assessment of risk of bias

Each study will be given a risk of bias assessment by two independent authors. Where there is disagreement, the same means of resolution will be used as was applied to data extraction. Bias assessment will follow both the International Cochrane Collaborations [18] recommendations in addition to the Newcastle-Ottawa Scale for observational studies [24]. The former assesses bias in selection, performance, detection, attrition and reporting. It has a three-point scale rating each area and the overall study as having a high, low or unclear risk of bias. The latter assesses comparability and outcome in observational studies. For non-observational non-randomised studies that are not amenable to the Newcastle-Ottawa Scale, bias will be assessed with the use of the Risk Of Bias In Non-randomised Studies-of Interventions (ROBINS-I tool) [25]. The Cochrane Collaboration developed this tool as to provide a comprehensive seven-domain assessment of bias.

### Data synthesis

A narrative description of the findings will be provided. This will focus on:

- The population studied
- The type of intervention/diagnostic strategy
- The reported prevalence of Lynch syndrome.

In addition, and where possible, secondary outcomes will also be described including the mutational distribution between the four MMR genes and the pathological characteristics of Lynch syndrome-related disease and non-Lynch syndrome-related disease.

It is assumed the data will have a high degree of heterogeneity. This will be described using the appropriate statistical means, namely the Cochran’s Q, and described by the *I*^2^ statistic. Where the heterogeneity is found to be > 60%, *P* < 0.10 via *I*^2^ analysis, a weighted average will not be performed. The type of participants and methodological differences will be examined as to explore the level of the heterogeneity. Furthermore, subgroups will be analysed in an attempt to decrease the heterogenetic effects.

Qualitative synthesis will be performed looking to the medians alongside the interquartile ranges of proportions reported in included studies.

### Statistical analysis

To estimate the prevalence of Lynch syndrome in women with endometrial cancer, a pooled proportion meta-analysis will be conducted using the number of events and number of observations in each study. All statistical analyses will be performed in R version 3.4.1 using the ‘metaprop’ function, which is part of the ‘meta’ package. An overall pooled proportion will be calculated by the back transformation of the weighted mean of the logit transformed proportions, using a random effects model; results will be presented as a pooled proportion with 95% CI. The degree study heterogeneity will be assessed by evaluation of the *I*^2^ score, with an *I*^2^ score of > 75% indicating significant study heterogeneity.

## Discussion

This systematic review looks to collate data from individual studies in order to determine robust estimates of the prevalence of Lynch syndrome in women with endometrial cancer. The information provided by this review will enable healthcare providers to make decisions regarding the screening of selected or unselected populations of women with endometrial cancer for Lynch syndrome. Furthermore, it will explore the impact of pre-selection and the diagnostic screening test used. These data have the potential to change and direct clinical management.

This systematic review has been registered on PROSPERO (ref CRD42017081707) and will be published in the peer-reviewed literature.

## Abbreviations

MMR: Mismatch repair
MSI: Microsatellite instability
MSI-H: Microsatellite instability-high
